# Spatial Distribution and Contamination Assessment of Surface Heavy Metals off the Western Guangdong Province and Northeastern Hainan Island

**DOI:** 10.3390/ijerph15091897

**Published:** 2018-08-31

**Authors:** Qian Ge, Zuo George Xue, Fengyou Chu

**Affiliations:** 1 Second Institute of Oceanography, State Oceanic Administration, Hangzhou 310012, China; chu@sio.org.cn; 2 Key Laboratory of Submarine Geosciences, State Oceanic Administration, Hangzhou 310012, China; 3 Department of Oceanography and Coastal Sciences, Louisiana State University, Baton Rouge, LA 70803, USA; zxue@lsu.edu; 4 Center for Computation and Technology, Louisiana State University, Baton Rouge, LA 70803, USA; 5 Coastal Studies Institute, Louisiana State University, Baton Rouge, LA 70803, USA

**Keywords:** surface sediments, heavy metals, South China Sea, pollution degree, potential ecological risk, sources identification

## Abstract

Surface sediments collected from the continental shelf off the western Guangdong Province and northeastern Hainan Island are analyzed for selected heavy metals contents including Cd, Cr, Cu, Pb, Zn, and As to determine spatial distribution, potential ecological risks, and sources. In addition, some of the controlling factors of heavy metals distribution are also discussed. The average heavy metals contents decrease in the order of Zn > Cr > Pb > Cu > As > Cd. The averaged pollution degree, as shown by the index of geo-accumulation (*I_geo_*), decreases in the order of Zn > Cu > Pb > Cr > Cd > As. Due to the barrier of islands, the *I_geo_* values of Zn, Pb, Cr, Cu, and Cd near the Hailing and Xiachuan Islands are larger than those in other areas. Meanwhile, the *I_geo_* value of As near the coastal area off the estuary of Wanquan River is clearly larger than that in other areas. Based on the results of potential ecological risk index, Cd, Cu, and As should be paid more attention for the contamination risk in future. The results of Pearson correlation analysis and principal component analysis indicate that Zn, Cr, Pb, Cu, and Cd are mainly from the Pearl River and surrounding small rivers, whereas As originates from the Hainan Island. The grain size is one of the main controlling factors for heavy metals distribution, and the anthropogenic activity also plays an important role.

## 1. Introduction

Over the past century, because of the rapid industrialization, estuaries and coastal areas have become the main sinks of the anthropogenic pollutants, which mainly consist of industrial and domestic sewage discharges, smelting, mining, and *e*-wastes recycling. Toxic contaminants such as persistent organic pollutants and metals bring increasing pressure to the coastal and estuarine ecosystems over the past decades due to the enhanced human activities [1]. Metals are naturally occurring constituents in the environment and vary in concentrations in different areas. Unlike organic pollutants, metals are considered as non-degradable pollutants, especially for the heavy metals. After deposition, the chemical and biological processes may allow heavy metals to be desorbed from surface sediments upon which they are released into the water column [2]. Therefore, marine sediments serve as both sinks and potential secondary sources of heavy metals [3]. The research on heavy metals in surface sediments provides significant insights into the metal pollution in aquatic systems. In recent years, numerous efforts have been made to investigate the influences of heavy metals in the coastal area [4,5,6,7,8,9]. All results show the influences of terrigenous input for the heavy metal pollution.

The continental shelf in the northern South China Sea is relatively flat and wide, and receives a large amount of materials from the Pearl River and the surrounding land, which is one of the most developed regions in China. The multiple sources and hydrodynamic conditions make it play an important role in discussing the sources, transportation and deposition of heavy metals in the South China Sea. However, the comprehensive understanding of heavy metals provenance and their potential threats for the marine ecosystem in this area is limited.

The main objectives of the present study are to (1) characterize the spatial distribution of heavy metals in surface sediments, (2) identify the sources of heavy metals and controlling factors of the heavy metals distribution, and (3) assess the heavy metals pollution degree and their potential ecological risk index (PERI).

## 2. Materials and Methods

### 2.1. Study Area

**Figure 1:**
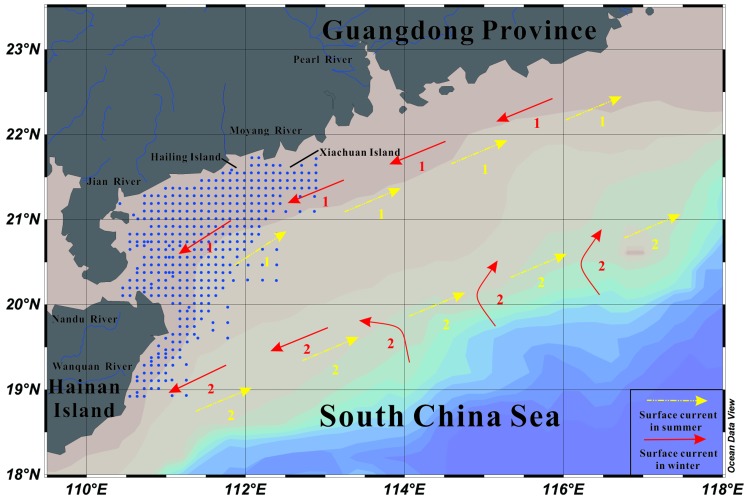
Map of the study area showing the locations of surface sediment samples. Seasonal variations of surface circulation patterns on the northern South China Sea continental shelf (revised from reference [13]). 1. Chinese Coastal Current; 2. South China Sea warm current.

The study area is located on the northern South China Sea continental shelf, off the western Guangdong Province and northeastern Hainan Island (Figure 1). Annual sediment fluxes in this area include approximately 84 Mt from Pearl River and 4 Mt from other small rivers (e.g., Jian River, Moyang River, Nandu River, and Wanquan River) [10]. The general surface circulation in the study area is largely seasonal and is driven primarily by the distinct seasonal monsoon winds [11,12]. In winter, the Guangdong coastal current flows along the northeasterly direction of the winter monsoon winds. In summer, the South China Sea warm current becomes prevailing southeasterly surface current forced apparently by the summer monsoon winds [13] (Figure 1).

### 2.2. Sample Collection and Analytical Methods

A total of 388 surface sediment samples were collected by the Second Institute of Oceanography, State Oceanic Administration and South China Sea Branch, State Oceanic Administration in 2008 (samples location see Figure 1). The heavy metals contents of Cd, Cr, Cu, Pb, Zn, and As were identified using an Inductively Coupled Plasma Mass Spectrometer (ICP-MS) (iCAP Qc, Thermo Fisher Scientific, Waltham, MA, USA) at the Institute of Geophysical and Geochemical Exploration, Chinese Academy of Geological Sciences, China. Prior to chemical analysis, the samples were dried below 105 °C, crushed to <200 mesh size in an agate mortar, and stored in clean polyethylene bags at room temperature. For acid digestion, precisely 0.25 g of each sample was put in a Teflon bomb with an acid mixture (5:4:1 V (HNO_3_) + V (HCl) + V (HF)) [14] and then heated to 120 °C for 12 h on a heating plate. The acid digestion was repeated until only a negligible amount of white residue remained. Then, the solution was evaporated to dry and extracted with HNO_3_. There were 22 certified reference materials and 23 duplicate samples analyzed for the data quality control. Most of the mean relative standard deviations for duplicate samples are less than 7.00%, except for Cd (10.62%).

### 2.3. Assessment Methods

#### 2.3.1. Index of Geo-Accumulation (*I_geo_*)

The *I_geo_* was firstly introduced by Müller [15]. It is a quantitative index to study heavy metal contamination in aquatic sediments and has been widely used to evaluate the contamination degree of heavy metals in surface sediments. The formula was as follows:

(1) $$\;I_{geo\;}=log_{2}\left(\frac{C^{i}}{1.5\times C_{n}^{i}}\right)$$

where $C^{i}$ is the mean content of the heavy metal *i* in the surface sediments; $C_{n}^{i}$ is the baseline content of *i*. The factor 1.5 is used due to possible variations in background values for the metal in the environment [16]. The baseline values ($C_{n}^{i}$) of Cu, Pb, Zn, Cr, Cd, and As around the study area are 14.2 μg/g, 28.8 μg/g, 59 μg/g, 56 μg/g, 0.08 μg/g, and 13.5 μg/g, respectively, which are obtained through analyzing the soils from Guangdong Province [17]. The *I_geo_* values are grouped into classes to represent different pollution levels, the definitions [18] for which are listed in Table 1.

#### 2.3.2. PERI

The *I_geo_* can only reflect the influence of human activities on the enrichment of single heavy metal and do not consider the bioavailability, hydrodynamic condition or combined effects of heavy metals [19]. Therefore, the PERI was a quantitative index that evaluated the pollution and potential risks associated with the accumulation of one or more heavy metals [20,21]. The formulas of PERI were as follows:

(2) $$\;C_{dg\;}=\overset{k}{\underset{i=1}{\sum }}C_{f}^{i}=\overset{k}{\underset{i=1}{\sum }}\frac{C^{i}}{C_{n}^{i}}$$

(3) $$\;\mathrm{RI}=\overset{k}{\underset{i=1\;}{\sum }}E_{n}^{i}=\overset{k}{\underset{i=1}{\sum }}\frac{T_{n}^{i}\cdot C^{i}}{C_{n}^{i}}$$

where $C^{i}$ is the mean content of the heavy metal *i* in the surface sediments; $C_{n}^{i}$ is the baseline content of *i*; $C_{f}^{i}$ is the contamination factor of *i*; $C_{dg}$ is the degree of contamination; $T_{n}^{i}$ is the toxic-response factor for *i*; $E_{n}^{i}$ is the potential ecological risk factor for *i*; RI is the PERI; and *k* is the species of the heavy metals. In this study, we use Cu, Pb, Zn, Cr, Cd, and As to evaluate the PERI. And the toxic-response factors ($T_{n}^{i}$) for these six elements are 5, 5, 1, 2, 30, and 10, respectively [20], the baseline values ($C_{n}^{i}$) are showed above. The categories [20] of pollution and PERI for sediments are listed in Table 2.

### 2.4. Statistical Methods

Multiple statistical analyses have been widely used to distinguish sources and controlling factors in heavy metal contamination assessments [3,6,22,23]. The Pearson correlation coefficients and principal component analysis among the various types of heavy metals were calculated and displayed in Table 3 and Table 4. In addition, the grain size data of the surface sediments from the study area have been published by Chu et al. [24] in 2010, we cite the mean grain size (Mz) data to compare with the contents of heavy metals in the statistical analyses. To maximize the sum of variances of squared loadings, a varimax rotation was used when calculating the principal components. The principal component analysis can reduce the dimensionality of geochemical data. Meanwhile, this method has been proven to be an effective tool that can be used to identify potential sources and controlling factors of heavy metals distribution and has been widely used in combination with correlation analysis [3,25].

## 3. Results and Discussion

### 3.1. Contents and Spatial Distribution of Heavy Metals in the Surface Sediments

Table 4 summarizes the basic statistics of heavy metals in surface sediments from the continental shelf off the western Guangdong Province and northeastern Hainan Island. The average contents of these elements decreased in the order of Zn (74.22 μg/g) > Cr (56.71 μg/g) > Pb (28.71 μg/g) > Cu (17.72 μg/g) > As (9.14 μg/g) > Cd (0.08 μg/g). The maximum values of Cd, Cu, and As exceed the secondary standard of Environmental quality standard for soils (GB 15618-1995) in China, and those of Cr, Pb, and Zn exceed the first standard (natural background). Based on the coefficient of variance, the contents of heavy metals varied distinctly among sampling sites, especially for Cu, As, and Cd, with the values of 64.88%, 52.67%, and 51.86%, respectively (Table 4). The coefficient of variance can reflect the transport ability for heavy metals with similar origins [7]. The higher coefficient of variance is associated with weaker transport regime. Therefore, the delivery of Cu is weakest while that of Cr is the strongest.

**Figure 2:**
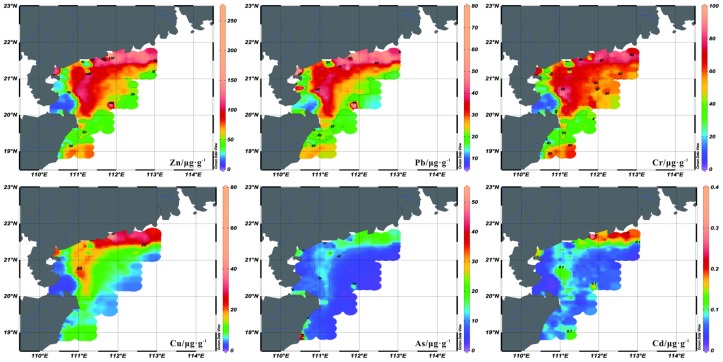
Spatial distribution patterns of the contents for heavy metals in the study area.

Ge et al. [26], based on geophysical data indicated that the Pearl River-derived fine sediments were mostly deposited in the water depth shallower than ~50 m on the continental shelf off the western Guangdong Province. As shown in Figure 2, the heavy metals exhibit various distribution patterns in the study area. For example, the content of Zn is rich in the water shallower than ~50 m, and then decreases to lower than 70 μg/g in deeper water. This spatial distribution pattern corresponds to those of silt and clay contents in the study area [24], which indicates that fine sediment is enriched of Zn. The distribution patterns of Pb, and Cr are similar to that of Zn, although their extensions are different. The distribution pattern of As shows slight variations in the study area, except for the shelf off the eastern Hainan Island. Meanwhile, the distribution patterns of Cu and Cd display the highest values in the coastal area off the Guangdong Province, around the Hailing, Xiachuan Islands (Figure 2).

### 3.2. Pollution Assessment

Heavy metals in marine sediments originate from various sources, primarily including the weathering of rocks, discharge of upstream wastewater and atmospheric input. Among these sources, wastewater is the dominant one in the coastal area [7]. Heavy metals are primarily transported to the ocean with terrigenous materials in either adsorbed or dissolved phase [27]. After delivered to the ocean, heavy metals can rapidly convert from the aqueous phase to the solid phase and ultimately accumulate in seafloor deposits [28]. Such accumulation can affect the environment and contaminate the marine food chain. Therefore, evaluating the harm of heavy metals’ pollution is critically important. 

**Figure 3:**
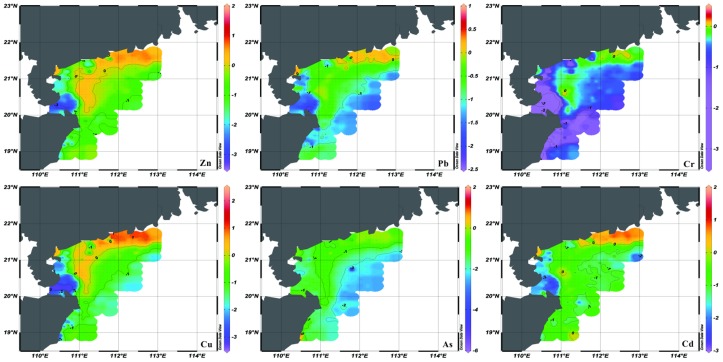
Spatial distribution pattern of index of geo-accumulation (*I_geo_*) for heavy metals in the study area.

The *I_geo_* values of heavy metals range from −2.89 to 1.72 with a mean value of −0.74 for Cd, −3.35 to 0.15 with a mean value of −0.71 for Cr, −3.34 to 2.39 with a mean value of −0.58 for Cu, −2.28 to 0.86 with a mean value of −0.69 for Pb, −3.47 to 1.51 with a mean value of −0.46 for Zn, and −5.78 to 1.32 with a mean value of −1.31 for As, respectively (Figure 3). The mean pollution degrees of these heavy metals can be ranked in the decreasing order of Zn > Cu > Pb > Cr > Cd > As. According to the classification of Fostner and Müller [18], the *I_geo_* values of Cr and Pb suggested no pollution or minor to moderate pollution (Figure 3, Table 1). However, the *I_geo_* values of Cd, Cu, Zn, and As implied heavier degrees of pollution (Figure 3). As shown in Figure 3, the *I_geo_* values of Zn, Pb, Cr, Cu, and Cd near the Hailing and Xiachuan Islands were larger than those in other areas, which could be attributed to the increasing of flocculation. Due to the block of these islands, the flow shear stress decreased, and the fluvial materials with heavy metals occurred flocculation effect to deposit easily. Meanwhile, the *I_geo_* value of As near the coastal area off estuary of Wanquan River was clearly larger than that in other areas (Figure 3). 

**Figure 4:**
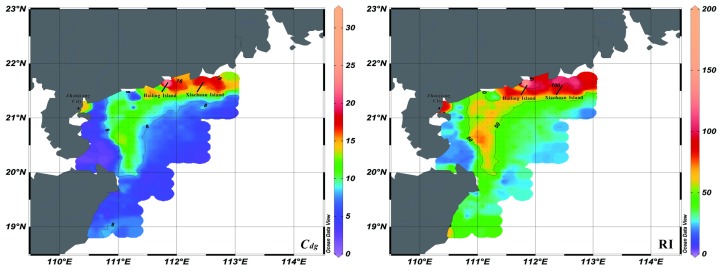
Spatial distribution patterns of the degree of contamination ($C_{dg}$) and potential ecological risk index (RI) for heavy metals in the study area.

Potential ecological risk related to heavy metals in surface sediments from the study area was calculated based on Equations (2) and (3). From Equation (2), the mean values of $C_{f}^{i}$ for Cu, Pb, Zn, Cr, Cd, and As are 1.25, 1.00, 1.26, 1.01, 1.00, and 0.68, respectively. The pollution degree caused by these elements can be ranked as follows: Zn > Cu > Cr > Pb = Cd > As. The mean value of $C_{dg}$ is 6.20, and the maximum value is 21.90. This implies that the pollution degree of these elements is low to high in most of the study area (Figure 4). Meanwhile, Figure 4 also shows that highly polluted regions are primarily located in the coastal area off the Guangdong Province, around the Hailing, Xiachuan Islands, and Zhanjiang City. From Equation (3), the mean values of $E_{n}^{i}$ for Cu, Pb, Zn, Cr, Cd, and As are 6.24, 4.98, 1.26, 2.03, 30.19, and 6.77, respectively. The potential ecological risk of these elements can be ranked as follows: Cd > Cu > As > Pb > Cr > Zn. The average value of RI is 51.47, and its maximum is 216.93. This implies that the potential ecological risks of these elements are low to moderate. Based on the comparison of these parameters, the rankings of potential ecological risk changed, with Cd and As rising and Zn falling. This shift indicates that Cd and As impose more potential ecological risks on the study area. The Figure 4 shows that moderate potential risk region is also found in the coastal area off the Guangdong Province and Hainan Island, especially for waters around Hailing and Xiachuan Islands.

### 3.3. Controlling Factors of Heavy Metals Distribution and Sources Identification

The Pearson correlation coefficients among the contents of heavy metals and Mz are shown in Table 3. The result showed that the heavy metals were significantly and positively correlated with each other except As. Content of As was not significantly correlated with any other heavy metals and Mz. Significant correlations were found between several elemental pairs: Cu-Cd (0.894), Pb-Cd (0.828), Zn-Cd (0.829), Cu-Cr (0.763), Pb-Cr (0.816), Zn-Cr (0.911), Pb-Cu (0.915), Zn-Cu (0.924), and Zn-Pb (0.931). Therefore, Cd, Cr, Cu, Pb, and Zn were positively correlated among each other and might have the similar sources.

The results of principal component analysis were shown in Table 5. The top two principal components explained 90.2% of the total variances. For factor loadings, F1 (53.5% of variance) shows high positive loadings of Cr, Pb, and Zn, and a moderate positive loading of Cu, and F2 (36.7% of variance) displays high positive loadings of As, Cd, and a moderate positive loading of Cu. Based on the combination patterns, these two principal components should represent different controlling factors.

**Figure 5:**
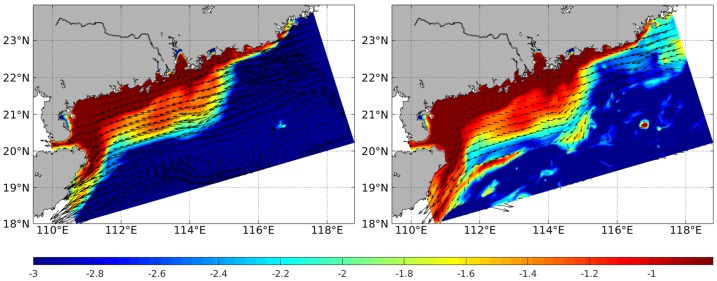
Simulated Suspended Sediment Concertation (SSC) in December 2016 (color shading scaled in log10, unit: g/L) and currents at surface (**left**) and bottom (**right**), respectively.

F1 represents the high positive loadings for Mz (Table 5). And the distribution patterns of silt and clay contents [24] are corresponding to those of Cr, Pb, Zn, and Cu (Figure 2), which means these heavy metals enrich in the fine sediments. Above evidence indicate that grain size is the most important factor controlling the distribution patterns of these heavy metals in the study area. Actually, the hydrodynamic environment could influence the characteristic of grain size [29]. As shown in Figure 3, the mean pollution degrees of these heavy metals are mostly moderate, especially for the nearshore area. And the distribution patterns of these heavy metals show agreements with that of Pearl River-derived fine sediment [26], indicating the possibility of Pearl River source. To further validate this transport pattern, we refer to the suspended sediment concentration (SSC) in winter simulated by the numerical model (Figure 5). This model adopts the Regional Ocean Modelling System (ROMS), which is a free-surface, finite difference circulation model, and is formulated in a vertical terrain following sigma coordinate [30]. Model simulated fine sediments confirmed the transport pattern revealed by the heavy metal data in this study. Yang et al. [31] proposed that the fluvial sediments in the Yellow and East China Seas were firstly deposited on the continental shelf near the estuary, and then re-suspended and transported by the southwestward Chinese coastal current when East Asian winter monsoon prevails. This sediment transportation pattern of “summer deposit and winter transport” is also proved in the northern South China Sea [26]. The Pearl River-derived fine sediment carried with Cr, Pb, Zn, and Cu transported into the ocean, and deposited on the shelf near the estuary firstly in summer. When winter monsoon prevails, these sediments were re-suspended and transported by the southwestward Chinese Coastal Current toward the Leizhou Peninsula. The high SSC area locates in the water shallower than ~50 m (Figure 5). The sediment transport mechanism can explain the spatial distribution of Cr, Pb, Zn, and Cu, which decreased from north to south and west progressively. Meanwhile, the anomalously high contents and *I_geo_* around the Hailing and Xiachuan Islands could be explained by the blocking of the Pearl River-derived sediments (Figure 2 and Figure 3).

Another important factor affecting the spatial distribution of heavy metals is anthropogenic activity. F2 displays high positive loadings of As, Cd, and a moderate positive loading of Cu. The top three heavy metals of high potential ecological risk are Cd, Cu, and As, which shows the influence of anthropogenic activity. The distribution pattern of As was different from that of other heavy metals, with higher contents on the shelf off the eastern Hainan Island (Figure 2). Meanwhile, the distribution pattern of RI also showed a high value region in this area, which was different to that of $C_{dg}$. The rankings of potential ecological risk showed the rising threat of As. This area was mainly influenced by the local rivers from eastern Hainan Island, such as Wanquan River. Although the pollution degree in this area is low right now, the potential ecological risk is rising due to the rapid developing of nearby cities, such as Town of Boao (Figure 4). Through analyzing the distribution patterns of Cu and Cd (Figure 2), we consider that the main sources of these heavy metals are rivers from Guangdong Province. The Pearl River flows through Guangdong Province, which is one of the most developed regions in China. The rapid economic development brings lots of pollutions, especially for Cu and Cd. In addition to the Pearl River, other sources might come from the mining and electroplating industry along some local rivers, such as Moyang River [32]. In conclusion, the anthropogenic activity makes the potential ecological risks of As, Cd, and Cu rising, and the weak delivery capacity of these heavy metals may aggravate the pollution.

## 4. Conclusions

We analyzed the spatial distribution patterns, *I_geo_*, PERI, controlling factors, and sources of heavy metals including Cd, Cr, Cu, Pb, Zn, and As in the surface sediments from the continental shelf off the western Guangdong Province and northeastern Hainan Island. The average contents of Zn, Cr, Pb, Cu, As, and Cd were 74.22, 56.71, 28.71, 17.72, 9.14, and 0.08 μg/g, respectively. Meanwhile, Zn, Cr, Pb, and Cu exhibited similar spatial distribution patterns decreasing from north to south and west toward the Qiongzhou Strait, which corresponded to the pathway of the Pearl River-derived fine sediments. Based on the results of *I_geo_* and PERI, Cd, Cu, Zn, and As were identified as the major heavy metal pollutants in surface sediments. The potential ecological risks of heavy metals are low to moderate. The results of statistical analyses showed that Zn, Cr, Pb, Cu, and Cd were mainly from the Pearl River and surrounding small rivers, whereas As originated from the Hainan Island. The factors controlling heavy metals characteristics were analyzed and indicated that grain size is one of the main factors. In addition, the anthropogenic activity can also influence the distribution patterns of heavy metals.

## Figures and Tables

**Table 1:** Classification of Index of geo-accumulation (*I_geo_*) [18].

*I_geo_*	Class	Pollution Level
<0	0	Unpolluted
0–1	1	Unpolluted to moderately polluted
1–2	2	Moderately polluted
2–3	3	Moderately to strongly polluted
3–4	4	Strongly polluted
4–5	5	Strongly to very strongly polluted
>5	6	Very strongly polluted

**Table 2:** Classification of pollution degree and potential ecological risk for surface sediments [20].

$\;C_{dg\;}$	Degree of Contamination	RI	Grade of Potential Ecological Risk to the Environment
<6	low pollution	<150	low potential risk
6–12	moderate pollution	150–300	moderate potential risk
12–24	high pollution	300–600	high potential risk
≥24	severe pollution	≥600	severe potential risk

**Table 3:** Pearson correlation matrix of heavy metals contents and mean grain size [24].

	As	Cd	Cr	Cu	Pb	Zn	Mz
As	1						
Cd	0.594 **	1					
Cr	0.347 **	0.651 **	1				
Cu	0.599 **	0.894 **	0.763 **	1			
Pb	0.626 **	0.828 **	0.816 **	0.915 **	1		
Zn	0.492 **	0.829 **	0.911 **	0.924 **	0.931 **	1	
Mz	0.168 **	0.571 **	0.845 **	0.630 **	0.688 **	0.790 **	1

** Correlation is significant at the 0.01 level (2-tailed).

**Table 4:** General characteristics of the heavy metals contents in surface sediments from the study area.

Parameter	Cd	Cr	Cu	Pb	Zn	As
Maximum (μg/g)	0.40	93.50	111.80	78.10	251.90	50.55
Minimum (μg/g)	0.02	8.20	2.10	8.90	8.00	0.37
Average (μg/g)	0.08	56.71	17.72	28.71	74.22	9.14
Coefficient of variance (%)	51.86	35.53	64.88	36.73	45.41	52.67

**Table 5:** Rotated component matrix for principal component analysis loadings for heavy metals contents and mean grain size [24] in the surface sediment from the study area.

Component	F1	F2
Cd	0.580	**0.701**
Cr	**0.912**	0.268
Cu	**0.677**	**0.678**
Pb	**0.716**	0.645
Zn	**0.848**	0.499
As	0.045	**0.938**
Mz	**0.942**	0.053
Variance %	53.5	36.7
Cumulative variance %	53.5	90.2

Bold fonts represent the high positive loadings in principal component analysis.
